# Serum Proteins Enhance Dispersion Stability and Influence the Cytotoxicity and Dosimetry of ZnO Nanoparticles in Suspension and Adherent Cancer Cell Models

**DOI:** 10.1186/s11671-015-1158-y

**Published:** 2015-11-17

**Authors:** Catherine B. Anders, Jordan J. Chess, Denise G. Wingett, Alex Punnoose

**Affiliations:** Department of Physics, Boise State University, Boise, ID 83725 USA; Biomolecular Sciences PhD program, Boise State University, Boise, ID 83725 USA; Department of Biological Sciences, Boise State University, Boise, ID 83725 USA; Department of Physics, University of Oregon, Eugen, OR 97403 USA

**Keywords:** Zinc oxide nanoparticles, Dispersion stability, Nanotoxicity, Dosimetry

## Abstract

Agglomeration and sedimentation of nanoparticles (NPs) within biological solutions is a major limitation in their use in many downstream applications. It has been proposed that serum proteins associate with the NP surface to form a protein corona that limits agglomeration and sedimentation. Here, we investigate the effect of fetal bovine serum (FBS) proteins on the dispersion stability, dosimetry, and NP-induced cytotoxicity of cationic zinc oxide nanoparticles (nZnO) synthesized via forced hydrolysis with a core size of 10 nm. Two different in vitro cell culture models, suspension and adherent, were evaluated by comparing a phosphate buffered saline (PBS) nZnO dispersion (nZnO/PBS) and an FBS-stabilized PBS nZnO dispersion (nZnO – FBS/PBS). Surface interactions of FBS on nZnO were analyzed via spectroscopic and optical techniques. Fourier transformed infrared spectroscopy (FTIR) confirmed the adsorption of negatively charged protein components on the cationic nZnO surface through the disappearance of surfaced-adsorbed carboxyl functional groups and the subsequent detection of vibrational modes associated with the protein backbone of FBS-associated proteins. Further confirmation of these interactions was noted in the isoelectric point shift of the nZnO from the characteristic pH of 9.5 to a pH of 6.1. In nZnO – FBS/PBS dispersions, the FBS reduced agglomeration and sedimentation behaviors to impart long-term improvements (>24 h) to the nZnO dispersion stability. Furthermore, mathematical dosimetry models indicate that nZnO – FBS/PBS dispersions had consistent NP deposition patterns over time unlike unstable nZnO/PBS dispersions. In suspension cell models, the stable nZnO – FBS/PBS dispersion resulted in a ~33 % increase in the NP-induced cytotoxicity for both Jurkat leukemic and Hut-78 lymphoma cancer cells. In contrast, the nZnO – FBS/PBS dispersion resulted in 49 and 71 % reductions in the cytotoxicity observed towards the adherent breast (T-47D) and prostate (LNCaP) cancer cell lines, respectively. Presence of FBS in the NP dispersions also increased the reactive oxygen species generation. These observations indicate that the improved dispersion stability leads to increased NP bioavailability for suspension cell models and reduced NP sedimentation onto adherent cell layers resulting in more accurate in vitro toxicity assessments.

## Background

When prepared in nanostructured form, many materials develop novel and unique physicochemical properties leading to their use in nanotechnology applications such as quantum dots and MRI contrast agents for imaging [1], biosensors that use surface plasmon resonance [2], and nanomedicinal approaches that employ hyperthermia and photothermal therapy [3]. Efficient use of NPs and nanocarriers in biomedical applications, such as delivery of therapeutic and imaging agents into tumor sites and genetic materials into cells, has been successfully demonstrated in recent years [4–6]. Interestingly, cytotoxicity is a property that is inherent in many of these commonly used materials when they are synthesized with their crystallite size in the nanoscale range [7–9]. This has raised significant scientific and public concerns on the environmental and health effects of every nanomaterial before employing them in nanotechnology applications. It is therefore necessary to evaluate the cytotoxic response of nanomaterials and the underlying mechanisms of nanomaterial toxicity to accurately address the growing concerns about the impact of the unwanted cytotoxicity of nanomaterials.

Numerous studies reported in the literature have shown that NP-induced toxicity depends on the nanomaterial properties such as crystallite size [10, 11], shape [12], charge [13], and chemical phase [14]. Some studies have also shown that the toxic response of a given nanomaterial might vary with the specific cell/organism type such as prokaryotes versus eukaryotes [15], gram-positive bacteria versus gram-negative bacteria, and normal cells versus cancerous cells [16]. These observations suggest that the role of organism-specific factors such as surface structure of cells [17, 18] and the proliferation status [10, 16] also influence the toxic response. Although the cytotoxicity of nanomaterials is an unfavorable feature for many applications, it could become very useful for certain therapeutic applications, if the toxicity is cell- or organism-specific. The cytotoxicity reported for most nanomaterials does not display a cell-specific or organism-specific behavior. However, many groups [16, 19, 20], including the authors, have demonstrated that certain ZnO NP formulations show a strong preferential cytotoxicity to cancer cells of identical lineage. For example, while resting primary human immune cells (T lymphocytes) showed no appreciable effect when treated with ≤5 mM concentrations of ZnO NPs, cancer cells of the same lineage showed significantly increased NP-induced toxicity resulting in approximately 28–35-fold increases in NP sensitivity [10, 16]. Thus, toxicity studies are extremely important both to assess the environmental and health impacts of NPs and to modify the nanomaterial design to make them safer, as well as to develop novel nanomedicinal approaches utilizing cell-specific cytotoxicity.

Considering the large number and types of new nanomaterials being developed and coming to the market, in vitro studies are essential in assessing the toxicity in high-throughput systems as they provide rapid and cost-effective screening. In most in vitro cytotoxicity experiments, cells are cultured in a culture medium to which a prepared dispersion of the NPs in a suitable solution is introduced. In contrast, in vivo toxicology studies frequently employ NPs dispersed in a suitable medium that are subsequently injected into the blood vessels or tissues of the animal model. If successful, the dispersed NPs will reach tumor sites and achieve deep penetration and entrapment in the cancerous tissue by means of the enhanced permeability and retention (EPR) effect, characterized by increased vascular permeability and compressed lymphatic networks in solid tumors [21]. However, making highly stable and efficient dispersions of nanoparticles to achieve efficient nanoparticle delivery for both in vitro and in vivo applications has been a challenging task. A major difference that NPs possess compared to the well-studied conventional toxic chemicals (which are well soluble molecules and ions) is the difficulty in dispersing them in suitable solutions and culture media and to maintain them as stable dispersions for sufficient durations [22]. For most in vitro studies, NP suspensions should remain stable at least for 24 h while in vivo studies require stability for much longer durations [23]. Formation of large agglomerates and their sedimentation over longer periods often prevents a significant fraction of the NPs from participating in efficient interactions with the test cells and the resulting cytotoxic response.

Nanoparticle dispersion stability is a complex interplay between the agglomerate size of NP complexes in dispersion (hydrodynamic size) and their sedimentation behavior over time [24]. Factors that affect NP stability include NP size [25, 26], surface structure [18, 27], capping agents [28], pH [29], ionic strength [29], organic and protein components [26, 27, 30], and free ions in the solution system [31]. Although it is now known that the physicochemical properties of the nanomaterial, the specific cell/organism type, and the membrane properties [10, 17, 18] of the test organism play a crucial role in the resulting cytotoxic response, very few studies have focused on the important role of NP dispersion characteristics and dispersion stability and the effects of NP agglomeration and sedimentation in the resulting toxicity [32, 33]. Furthermore, Cho et al. [34] have recently shown that the cellular uptake of gold NP in breast cancer cells measured using conventional upright in vitro cell culture configuration was significantly higher than that obtained when an inverted setup was used, suggesting the effect of NP sedimentation and diffusion in the media play a significant role in the NP-cell interaction. In addition, suspension stability has been shown to directly influence effective administered dose of NPs to adherent cells grown in vitro [22, 32, 33].

When NPs are introduced to biological fluids such as human plasma, a biomolecular corona consisting of proteins and small molecules rapidly forms on the NP surface [27, 35–37]. Since the blood circulatory system is the most probable treatment administration option for NPs into the human body, it is important to investigate how adsorption of blood proteins on NPs will affect the cellular response, and more importantly, the differential cytotoxicity of ZnO NPs between cancerous and normal cells. The effect of having serum in culture medium and/or adsorption of the serum proteins on some NP systems such as silica [38], carbon nanotubes [39], graphene oxide [40], and ZnO [41, 42] has been studied recently. In all these cases, the primary effects of serum addition included improved dispersion stability and a clear reduction of the NP-induced cytotoxicity. Interestingly, the abovementioned studies were conducted using adherent cell models; therefore, comprehensive studies are needed to determine if similar responses are present for suspension cell models as well. Hypothetically, increases in the NP-induced toxicity in suspension cell models would be observed as NP sedimentation decreases, thereby improving the bioavailability of the NPs to the cells.

In this study, we investigated the use of FBS as a ZnO NP coating agent and the effects of this FBS coating the surface structure of the uncoated NPs, the dispersive stability of the NPs in solution, and the effective dosimetry of the NPs in cellular toxicity assays. Since NP dispersion stability and dosimetry is highly dependent upon a balance of NP diffusion and sedimentation velocities within a solution [32–34, 43, 44], the use of multiple suspension and adherent cell models was necessary to fully characterize any observations attributable to the addition of the FBS. A complete NP characterization profile was employed to fully interrogate NP surface changes as a result of the FBS coating. In addition, we examined the agglomeration and sedimentation behavior of the FBS-coated ZnO NPs (nZnO – FBS) compared to uncoated ZnO NP (nZnO) in biologically relevant solutions. Finally, we investigated the effects that FBS coating would have on the solubility of the nZnO, the NP-induced toxicity, and nZnO-induced reactive oxygen species production. As a whole, this work provides a comprehensive look at effects of FBS as a dispersion stabilizing agent in nanotoxicology studies.

## Methods

### Synthesis and Characterization of nZnO

The nZnO sample used in this study was produced using the forced hydrolysis of Zn acetate dehydrate precursor in diethylene glycol (DEG). The solution was held at 150 °C for 90 min. Nanopure water was added to the solution at 80 °C to obtain the desired crystallite size. This synthesis method allows for excellent control of the crystallite size by carefully regulating the temperature parameters and the hydrolysis ratio of the reaction media [10]. Once cooled to room temperature, the nZnO were separated from solution via centrifuging at 20,000 rpm and subsequent washings with ethanol. A dried FBS-coated (nZnO – FBS^dried^) sample was prepared by sonicating the nZnO with FBS for 10 min. The resulting suspension was then centrifuged, and the resulting pellet rinsed several times with nanopure water to remove any unbound FBS. The pellet was dried overnight at 60 °C to create a powdered nZnO – FBS^dried^ form for material characterization. Both NP samples were thoroughly characterized and investigated in detail using X-ray diffraction (XRD), transmission electron microscopy (TEM), zeta potential measurements, UV-vis spectrophotometry, and Fourier transformed infrared spectroscopy (FTIR). X-ray diffraction (XRD) spectra were recorded at room temperature on a Philips X’Pert X-ray diffractometer with a Cu K_α_ source (*λ* = 1.5418 Å) in Bragg-Brentano geometry. The loose powder samples were leveled in the sample holder to ensure a smooth surface and mounted on a fixed horizontal sample plane. Lattice parameters and crystal size were analyzed with Rietveld refinement using Materials Analysis Using Diffraction (MAUD) software, corrected for instrumental broadening [45]. TEM and high-resolution TEM analysis was carried out using an FEI Tecnai and FEI Titan, respectively. The operating voltage of the Tecnai microscope was 120 and 300 kV on the Titan. Image processing was carried out using the Digital Micrograph software from Gatan (Pleasant, CA, USA). Room-temperature optical spectra in the ultraviolet and visible light wavelength ranges were collected using a CARY 5000 spectrophotometer. Zeta potentials of the powdered samples of nZnO were measured in nanopure water as a function of pH with a Malvern Zetasizer NanoZS. The temperature was equilibrated to 25 °C, and the pH was varied in the 6 to 12 range using 1.0 N HCl and 1.0 N NaOH prior to collecting the data. At least six data collections per run were performed on three separate aliquots of the ZnO suspension for each sample. The Zetasizer unit was also used to measure the hydrodynamic size of nZnO aggregates dispersed in selected media. Several solution systems were used for NP stock preparation and analyzed during this experiment.

### NP Stock Preparation

To achieve the most stable dispersion for each solution, the critical material-specific delivered sonication energy (DSE_cr_) for the ZnO NPs was predetermined for the calorimetrically calibrated JSP Super-sonic bath sonication device employed for this study. A DSE_cr_ of 181 J/ml was established through established protocols outlined by Taurozzi et al. (2011) [46] and employed in multiple dosimetry studies [33, 47]. To achieve the needed DSE_cr_, ZnO NPs were suspended at concentrations of 25 mM and sonicated in the bath sonicator delivering a power of 1.05 W for a total 30 min. Several biologically relevant solutions were prepared per manufacturer’s instructions unless otherwise defined. They include nanopure water, PBS, FBS, Roswell Park Memorial Institute (RPMI) medium (RPMI 1640 buffered with sodium bicarbonate and Hepes, pH adjusted to 7.3), RPMI-based cellular medium (RPMI 1640 medium supplemented with 10 % FBS and 2 mM l-glutamine, 1.5 g/l sodium bicarbonate, 4.5 g/l glucose, 10 mM HEPES, 1.0 mM sodium pyruvate and penicillin-streptomycin), and DMEM-based cellular medium.

In addition to single solution NP dispersions, several NP stock dispersions were prepared with FBS. Based on most of the reported protocols found in the literature and our own preliminary data, 10 % FBS addition was found to provide optimal results and was used to prepare these stock dispersions. The stock dispersions involved first suspending the desired quantity of NPs in a volume of FBS equivalent to 10 % of the final solution volume and sonicating at 1.05 W for 10 min. After sonication, the FBS suspension was diluted to the calculated volume with the desired medium (90 % of the total volume) and re-sonicated at 1.05 W for an additional 20 min. These stock dispersions are referred to as either nZnO – FBS/water or nZnO – FBS/PBS for stocks prepared in nanopure water or PBS, respectively. Additionally, two nZnO – FBS formulations were created by removing the excess FBS prior to re-suspension and sonication in the appropriate solution. One formulation involved centrifuging the nZnO/FBS suspension after the initial sonication period followed by several washes with nanopure water to remove any unbound FBS. The remaining pellet was then re-suspended in the desired solution to the appropriate concentration to create nZnO – FBS^washed^/water or nZnO – FBS^washed^/PBS for nanopure water and PBS dispersions, respectively. Finally, stock dispersions for the dried sample, described in the previous section, are designated as nZnO – FBS^dried^/water and nZnO – FBS^dried^/PBS for nanopure and PBS dispersions, respectively.

### Extracellular Dissolution

For extracellular dissolution experiments, nZnO stock dispersions were prepared as previously described at 6 mM concentrations. To simulate cellular assay conditions, an aliquot of each stock dispersion was added to cellular media at a final nZnO concentration of 0.6 mM. All dispersions were stirred continuously to prevent nZnO sedimentation. At desired time points, an aliquot of the sample was removed and centrifuged at 5000×*g* for 20 min using an Amicon™ Ultra-4 Centrifugal Filter Unit with a 3-kD molecular weight cutoff (0.1 nm pore size) to remove any undissolved nZnO while allowing free zinc ions to pass through. Quantitative analysis of the dissolved Zn^2+^ ions was conducted on a Thermo X Series 2 quadrupole inductively coupled plasma mass spectrometer (ICP-MS) under normal operating conditions (i.e., no CCT) with the X_t_ cone set. Instrument performance was evaluated and optimized for each run. The instrument was calibrated using multi-element calibration standards containing Zn, Cr, Mn, Fe, Co, and Ni in 2 % HNO_3_ at concentrations of approximately 1, 10, 100, and 1000 ppb. Instrument drift was monitored and corrected using 20 ppb indium as an internal standard introduced online.

### Dosimetry Analysis and Empirical Deposition Fractions

Since NPs suspended in solution often form loosely packed agglomerates consisting of NP clusters and entrapped media, two empirical methods, the Harvard Volume Centrifugation Method (VCM) [32, 33] and the in vitro sedimentation, diffusion, and dosimetry (ISDD) computational model [43], were employed to determine the effective NP density of the agglomerates (*ρ*_e_, g/cm^3^), agglomerate porosity parameter (*ε*_agg_, unitless), diffusion rate (*D*, m^2^/s), sedimentation velocity (*V*, m/s), deposition factor, (α, h^−1^), and the deposition fraction curve [*f*_*D*_(*t*)]. Harvard VCM, which utilizes experimental methods to measure the effective volume of NP pellets (*V*_pellet_), was employed to estimate the *ρ*_e_ of the NP agglomerates. To measure the *V*_pellet_, NP suspensions were prepared at a concentration of 100 μg/ml in nanopure water and sonicated with 1.05 W of power for 30 min. The resulting stock dispersions were introduced to RPMI-based cellular media at a concentration of 1.25 mM and then centrifuged at 2000×*g* for 1 h in TPP (Techno Plastic Products, Trasadingen, Switzerland) packed cell volume tubes, and the resulting NP pellets were measured utilizing the TPP “easy read” measuring device by the same manufacturer.

Once the *V*_pellet_ for a NP dispersion was measured, the *ρ*_e_ of the NP agglomerates was then calculated using the following equation (DeLoid et al.) [32]:

1 $$ {\rho}_{\mathrm{e}} = {\rho}_{\mathrm{media}} + \left[\left(\frac{M_{\mathrm{ZnO}} - {M}_{\mathrm{ZnO}\mathrm{Sol}}}{V_{\mathrm{pellet}}SF}\right)\left(1-\frac{\rho_{\mathrm{media}}}{\rho_{\mathrm{ZnO}}}\right)\right] $$

This calculation represents the *ρ*_e_ as a function of the media density (*ρ*_media_) and density represented by the NP material. Since ZnO is partially soluble in nanopure water, the mass of solubilized ZnO (*M*_ZnOSol_) determined by ICP-MS as outlined above was subtracted from the original mass of ZnO (*M*_ZnO_). A theoretical stacking factor (SF) of 0.634 to approximate random close stacking employed by many dosimetry models [32, 33] was utilized. The density value for ZnO NPs (*ρ*_ZnO_) of 5.606 g/cm^−3^ was used. The deposition factor, (α) [43], a function of the sedimentation velocity (*V*), diffusion rate (*D*), and height of the media in the cell well (*L*)

2 $$ \alpha = \frac{D}{VL} $$

was then utilized to generate deposition fraction curves [*f*_*D*_(*t*)]:

3 $$ {f}_D(t)=1 - {e}^{-\propto t} $$

and time values needed to observe 90 % deposition of suspended NPs in solution (*t*_*90*_) [47]:

4 $$ t=\frac{- \ln \left(1-{f}_D(t)\right)}{\propto } $$

To model dosimetry kinetics for suspension cell models, the curves representing the fraction of available NPs [*f*_*A*_(*t*)] were created using the following equation:

5 $$ {f}_A(t)={e}^{-\propto t} $$

To determine the effective density, the in vitro sedimentation, diffusion, and dosimetry (ISDD) computational model employs the unitless agglomerate porosity parameter (*ε*_agg_)

6 $$ {\varepsilon}_{\mathrm{agg}}=1-{\left(\frac{d_{\mathrm{H}}}{d_{\mathrm{XRD}}}\right)}^{\mathrm{DF}-3} $$

where *d*_H_ represents the hydrodynamic size, *d*_XRD_ refers to the NP crystalline size measured by XRD, and DF is the specific fractal dimension. A DF value of 2.3, often employed to model metal oxide suspensions [47], was used in this study. Once *ε*_agg_ had been calculated, the *ρ*_*e*_ was calculated utilizing Eq. 7

7 $$ {\rho}_{\mathrm{e}}=\left(1-{\varepsilon}_{\mathrm{agg}}\right){\rho}_{\mathrm{ZnO}} + {\varepsilon}_{\mathrm{agg}}{\rho}_{\mathrm{media}} $$

The *ρ*_*e*_ value was then employed to calculate the sedimentation velocity (*V*, m/s) via Eq. 8

8 $$ V=\frac{g\left({\rho}_{\mathrm{ZnO}} - {\rho}_{\mathrm{media}}\right){d}_{\mathrm{H}}}{18\mu } $$

with *g* = 9.8 m/s^2^ and *μ* defined as the media viscosity (Pa · s). Finally, the diffusion rate (*D*, m^2^/s) was determined by

9 $$ D = \frac{RT}{3{N}_{\mathrm{A}}\pi \mu {d}_{\mathrm{H}}} $$

where *R* is the gas constant (*L* · kPa/K/mol), *N*_A_ is Avogadro’s number, and *T* (K) is the temperature.

### Cell Culture and Cytotoxicity Studies

For cell cytotoxicity assays, Jurkat T cell leukemia, Hut-78 T cell lymphoma, T-47D epithelial mammary gland carcinoma, and the LNCaP epithelial prostate cancer cell line (ATCC, Rockville, MD) were cultured in cellular medium. Additionally, the T-47D media was supplemented with 0.2 units/ml bovine insulin. Cells were maintained in log phase at 37 °C and 5 % CO_2_ and seeded at a concentration of 5 × 10^5^ cells/well in 96-well plates for Jurkat and Hut-78 cells. For the T-47D and LNCaP cells, the cells were first trypsinized using 0.25 % trypsin/0.53 mM EDTA (MP Biomedicals, LLC; Santa Ana, CA), re-suspended in fresh media, and then seeded at a concentration of 2.5 × 10^5^ cells/well in 24-well plates. The cells were then incubated overnight to allow the cells to re-adhere to the plate. Prior to treatment, the growth medium was gently aspirated from each well and replaced with 400 μL of fresh medium. Cells were subsequently treated with freshly sonicated (30-min) NPs reconstituted in the desired solution. Jurkat cells and Hut-78 cells were treated with NPs immediately after plating. For example, for a 2 mg/ml ZnO NP stock dispersion, 3.2 mg of NP was re-suspended in 1.58 ml of solution medium and sonicated at 50 W for 30 min. Then working dilutions were prepared from the NP stock dispersion and added to 200 μl of cell suspensions in 96-well plates or 400 μl of cell suspensions in 24-well plates and cultured for 24 h. After 24 h, the effects of the various NP stock dispersions on cell viability were determined using two different viability assays, alamar blue and flow cytometry. The alamar blue metabolic assay was employed to assess viability based on the fluorogenic redox indicator dye resazurin being converted to the fluorescent resofurin upon reduction by mitochondrial enzymes in metabolically active cells. In this assay, cell populations were seeded into the desired plate at the concentrations indicated above, treated with NPs for 20 h, and then incubated with alamar blue (10 % of total volume) for an additional 4 h. Changes in fluorescence were evaluated spectrophotometrically using excitation/emission at 530/590 nm in a Biotek Synergy MX® plate reader (Winooski, VT).

Flow cytometry was employed as an alternate viability assay to verify experimental results. NP-treated cells were dually stained with a fluorescein isothiocyanate-labeled anti-HLA ABC antibody and 50 μg/ml propidium iodide (PI) to monitor losses in membrane integrity. Fluorescent CountBright counting beads (Invitrogen, Carlsbad, CA) were added to samples to enable determinations of absolute cell numbers, and changes in PI staining were used to quantify cell death. Nanoparticles were excluded from analysis based on the absence of fluorescence signal and light forward scatter (FS) and side scatter (SSC) characteristics, and samples were analyzed using a BD FACS Caliber flow cytometer.

For CD4^+^ T cell cytotoxicity experiments, written informed consent was obtained from all blood donors and the University Institutional Review Board approved this study. Peripheral blood mononuclear cells were obtained from healthy human blood samples via Ficoll-Hypaque density centrifugation (Histopaque-1077, Sigma, St. Louis, MO) and CD4^+^ T cells isolated by negative immunomagnetic selection as previously reported [16]. Purified CD4^+^ T cells (>95 % purity) were cultured in RPMI/10 % FBS and suspended at a final concentration of 1 × 10^6^ cells/ml. CD4^+^ T cells were then incubated for 24 h with increasing amounts of each of the nZnO suspensions (24–800 μg/ml) in order to establish an IC_50_. CD4^+^ T cells were then stained and viability analyzed by flow cytometry.

### Reactive Oxygen Species (ROS) Detection

Mitochondrial superoxide levels were determined by flow cytometry using MitoSOX Red (Invitrogen, Eugene, OR) staining. MitoSOX Red is a cell permeable fluorescent dye that becomes oxidized by interacting with mitochondrial superoxide [48]. The resulting fluorescent signal occurs upon binding of the oxidized dye to nucleic acids. As a positive control, samples were treated with 0.2 nM of the mitochondrial electron transport inhibitor antimycin-A to ensure that the cells were capable of producing ROS. Jurkat cells were incubated with 0.4 mM nZnO or nZnO-FBS for 6, 18, and 24 h and then dually stained with CD3 antibody and 2.5 μM MitoSOX Red for 30 min. Mitochondrial superoxide levels were determined by flow cytometry.

### Statistical Analyses

Statistical analyses were performed using Jump Pro 10 software (SAS; Cary, NC). The cytotoxicity data were analyzed using repeated measures of variance with post hoc comparisons and significance levels defined as *p* < 0.05 to determine statistical differences between the means and allow within-sample variation to be separated from between-sample variation. Data for Figs. 10, 11, and 12 were analyzed using a two-way analysis of variance (ANOVA) to test for statistical significance of the model, and post hoc comparisons were used to test for statistically significant effects of treatment on cell viability (*p* < 0.05) with an asterisk denoting statistical significance in the indicated figures.

## Results and Discussion

### ZnO Nanoparticle Synthesis and Characterization

**Fig. 1:**
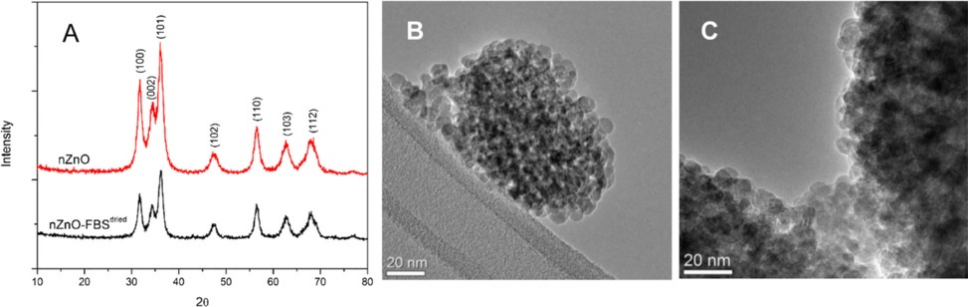
Crystal phase composition, size, and morphology characterization for nZnO and nZnO – FBS^dried^ NPs. **a** XRD spectra and TEM images for **b** nZnO and **c** nZnO – FBS^dried^

The pure ZnO NPs (nZnO) and the FBS-coated formulation (nZnO – FBS^dried^) were thoroughly characterized prior to use. Analysis of the powder X-ray diffraction spectra of the NPs revealed the expected hexagonal wurtzite crystal structure (Fig. 1). The MAUD software, which employs the Rietveld method [49] showed the particles had an average crystallite size of 10.0 ± 0.74 nm and lattice parameters *a* = 3.2580 Å and *c* = 5.226 Å. The nZnO – FBS^dried^ sample also showed similar crystallite size and lattice parameters suggesting that the FBS coating did not alter the crystalline structure of the nZnO nanoparticles. Additionally, TEM analysis confirmed that the NPs in both formulations were primarily spherical in morphology with similar size distributions (Fig. 1b, c).

**Fig. 2:**
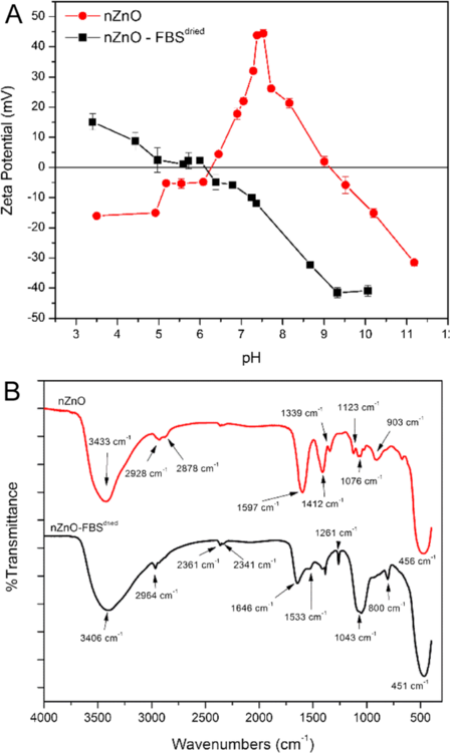
Surface property analysis for nZnO and nZnO – FBS^dried^. **a** Zeta potentials measured as a function of pH and **b** FTIR spectra with the wavenumbers of the main peaks marked

Surface charge for nZnO and the nZnO – FBS^dried^ NP samples, measured in nanopure water using electrophoretic measurements, was found to be 43.8 ± 0.55 mV and −11.8 ± 0.39 mV, respectively. Bovine serum albumin (BSA), the predominant component of FBS, has an isoelectric point (IEP) of 5.1 indicating that BSA would be positively charged at lower pH (<5.1) and negatively charged at higher pH values (>5.1) [50]. Therefore, the positive to negative shift in the zeta potential observed for the nZnO – FBS^dried^ NPs is indicative of the absorption of negatively charged protein components on the surface of the nZnO [51]. To determine the IEP of each NP suspension, the pH was varied from 3 to 12 using 0.1 M HCl or 0.1 M NaOH and the zeta potential was measured at several points in three independent trials (Fig. 2a). An IEP of 9.5 was observed for nZnO indicating these NPs would carry a positive charge below pH 9.5 and a negative charge at pH >9.5. In contrast, the nZnO – FBS^dried^ formulation had an IEP of pH 6.1, resulting in positively charged particles at much lower pH values (below 6.1) and negatively charged particles at pH >6.1. Below pH 6.5, partial NP dissolution was observed for nZnO but not for the nZnO – FBS^dried^ sample. The decreasing zeta potential trend observed at low pH values for nZnO has been documented for nZnO prepared using this synthesis method [28].

The FTIR spectra of nZnO and nZnO – FBS^dried^ are shown in Fig. 2b. Both samples contain a strong band characteristic of Zn-O vibrations [52] at 456 cm^−1^ (nZnO) and 451 cm^−1^ (nZnO – FBS^dried^). The nZnO sample exhibits two bands commonly associated with the carboxyl functional group, specifically 1412 cm^−1^ [ν_s_(COO^−^)] and 1597 cm^−1^ [ν_as_(COO^−^)] [53, 54]. Additionally, weaker bands consistent with τ(CH_2_) and ν(C–OH) at 903 and 1076 cm^−1^, respectively, indicate the presence of surface-adsorbed carboxyl groups originating from the zinc acetate precursor or the DEG solvent [52–54]. Furthermore, the absence of the ν(C=OOH) band at ~1720 cm^−1^ suggests that the carboxyl groups are in an expected deprotonated form as a result of the alkaline synthesis procedure [53]. Noticeably absent in the nZnO – FBS^dried^ samples are the strong symmetric and asymmetric stretching modes of the carboxyl group with new bands appearing in the spectrum at 1646 and 1533 cm^−1^ representing the amide I and amide II regions that arise from vibrations of the peptide bonds of the protein backbone [55, 56]. The amide I region, often used for secondary structure prediction, exhibits a maximum at 1650–1656 cm^−1^ when α-helices predominate in the protein secondary structure [56]. At 1646 cm^−1^, the amide I band of the nZnO – FBS^dried^ particles is close to this wavenumber maximum and suggestive of the presence of a protein with the α-helical structure components found in BSA. The slight shift of this maximum is consistent with a more random secondary structure (1644–1648 cm^−1^) and has been observed in protein adsorption and binding studies with albumin [57]. Taken together, these findings clearly establish the displacement of adsorbed carboxyl groups and strong binding of the FBS protein components [58, 59] on the nanoparticle surface.

### Agglomeration and Sedimentation Profiles

As previously discussed, determining the best media components and dispersion conditions for the preparation of stable nZnO dispersions was a major goal of this study. Reported cellular toxicity studies using Jurkat and Hut-78 cells have frequently employed a variety of different biological solutions used to disperse NP powder samples prior to treatment. Considerations in choosing stock solution components include maintaining a viable environment for the cells as well as creating a stable NP dispersion for treatment conditions. Commonly used stock solutions consist of nanopure water, PBS, or un-supplemented cellular media. In addition, various capping agents [60] or protein components are often utilized to reduce steric attractions between charged particles and solution components and reduce NP agglomeration [51, 60, 61]. Solutions such as PBS or un-supplemented media are generally preferred as they do not result in unwanted pH changes during the toxicity study or affect cellular osmolality. Likewise, media proteins such as BSA and FBS are frequently used as stabilizing agents.

**Fig. 3:**
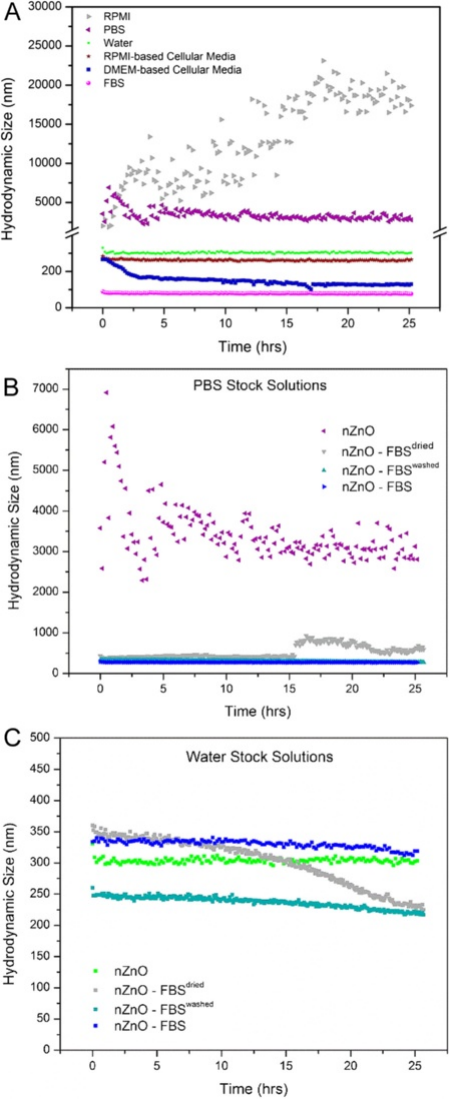
Aggregation profile evaluation for NP stock dispersions. Average hydrodynamic size profiles of 25 mM ZnO stock dispersions (pH = 7.3) for a 24-h time period. **a** nZnO in various biologically relevant solutions. **b** nZnO, nZnO – FBS^dried^, nZnO – FBS^washed^, and nZnO – FBS stock dispersions in PBS and **c** nZnO, nZnO – FBS^dried^, nZnO – FBS^washed^, and nZnO – FBS stock dispersions in nanopure water

The average hydrodynamic size of the nZnO sample in six different 25 mM stock dispersions was measured every 10 min for a 24-h time period using dynamic light scattering (DLS) technique (Fig. 3a). From this data, inferences can be made about the agglomeration tendencies of the NPs over time in different biological solution systems. Two of the solutions, RPMI and PBS, exhibited unstable average hydrodynamic size profiles over time evidenced by the immediate formation of very large agglomerates of 2031 and 3578 nm, respectively. The agglomerate sizes in PBS increased over time from 3578 to 5811 nm and eventually stabilized to a variable range of 2500–4200 nm over the remaining test period. In RPMI, the nZnO agglomerate size increased dramatically over time from 2031 nm to sizes exceeding the limits of the Zetasizer (~10,000 nm) by the end of the time period. These data indicate that agglomeration behavior of NP systems increases with increasing ionic strength of the dispersion solution (nanopure water < PBS < RPMI). All other test dispersions exhibit stable average hydrodynamic size profiles over time with average size profiles of 306 nm (nanopure water), 150–200 nm (DMEM-based cellular media), 263 nm (RPMI-based cellular media), and 78 nm (FBS). To evaluate the effect of FBS on dispersion stability, all three nZnO/FBS formulations, nZnO – FBS^dried^ (dried FBS-coated nZnO), nZnO – FBS^washed^ (FBS-coated nZnO with excess FBS removed), and nZnO – FBS (nZnO coated with 10 % FBS per volume of desired solution), were dispersed in both PBS and nanopure water at concentrations of 25 mM. For PBS stock dispersions, the FBS coating provided long-term hydrodynamic size stability (up to ~15 h) regardless of the formulation employed when compared to the nZnO/PBS dispersion (Fig. 3b). Beyond 15 h, the nZnO – FBS^dried^/PBS dispersions demonstrated some instability in agglomeration behavior with average agglomerate sizes increasing from approximately 400 to 800 nm. The addition of FBS in nanopure water dispersions did not appreciably change the agglomerate stability of the NP suspensions. Relatively stable agglomeration profiles were observed between ~200 and 350 nm for all dispersions (Fig. 3c). Interestingly, the nZnO – FBS^dried^ formulation also displayed a mild degree of dispersion instability at ~13 h. These findings, along with those for the PBS-based suspension, suggest that the nZnO – FBS^dried^ formulation is not the most ideal preparation for downstream applications which require long-term dispersion stability.

**Fig. 4:**
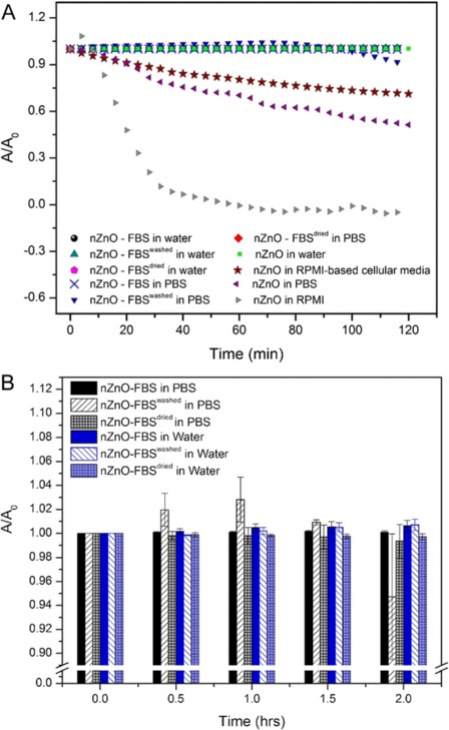
Sedimentation profile evaluation for NP stock dispersions. Sedimentation profiles of 25 mM ZnO stock dispersions (pH = 7.3) for a 2-h time period. **a** nZnO, nZnO – FBS^dried^, nZnO – FBS^washed^, and nZnO – FBS stock dispersions in various biologically relevant solutions. **b** Sedimentation histograms for 0, 0.5, 1.0, 1.5, and 2.0-h time points for nZnO – FBS^dried^, nZnO – FBS^washed^, and nZnO – FBS stock dispersions in PBS and nanopure water

Although average size profiles highlight the agglomeration tendencies of NP dispersions, they fail to depict how agglomerate formation affects NP sedimentation, changes in the net nZnO concentration in the solution over time, and the ever-changing size distribution of a complex NP dispersion. To address these shortcomings, experiments evaluating the sedimentation behavior of nZnO dispersions were performed by measuring changes in the optical absorbance intensity at *λ*_max_ (~378 nm corresponding to the band gap energy of nZnO) relative to the initial absorbance intensity. Decreases in the relative absorbance intensity at *λ*_max_ as a function of time have been correlated to decreasing NP concentrations in the solution as a result of NP sedimentation [23, 62]. The sedimentation tendencies over a 2-h time period for various stock dispersions are depicted in Fig. 4a. As expected, nZnO in RPMI rapidly settles out of the solution with 100 % sedimentation noted by the 1-h time point. Other dispersions that displayed sedimentation behavior were nZnO dispersions in both RPMI-based cellular media (29 % at 2 h) and PBS (49 % at 2 h). None of the nZnO – FBS formulations (nZnO – FBS^dried^, nZnO – FBS^washed^, or nZnO – FBS) showed any appreciable sedimentation behavior over the 2-h time period in PBS or nanopure water (Fig. 4a, b).

**Fig. 5:**
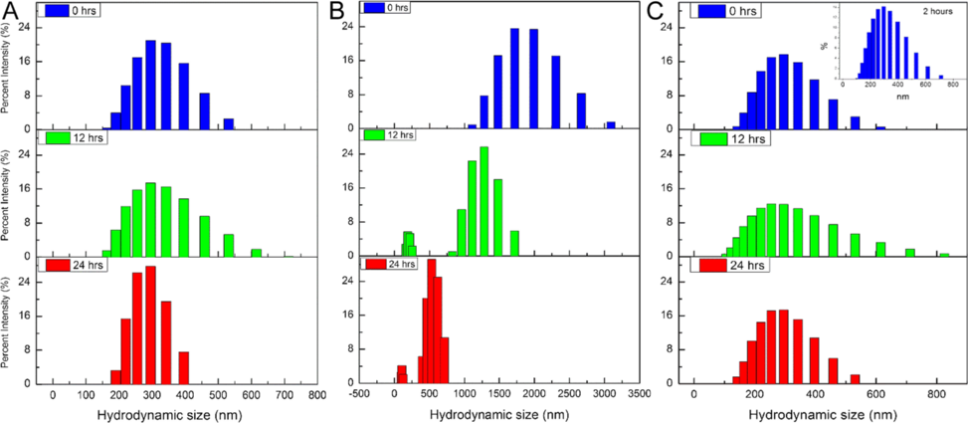
Hydrodynamic size distribution profiles at 0, 12, and 24 h. Histograms represent 25 mM stock dispersions prepared in **a** nanopure water, **b** PBS, and **c** RPMI-based cellular media

**Fig. 6:**
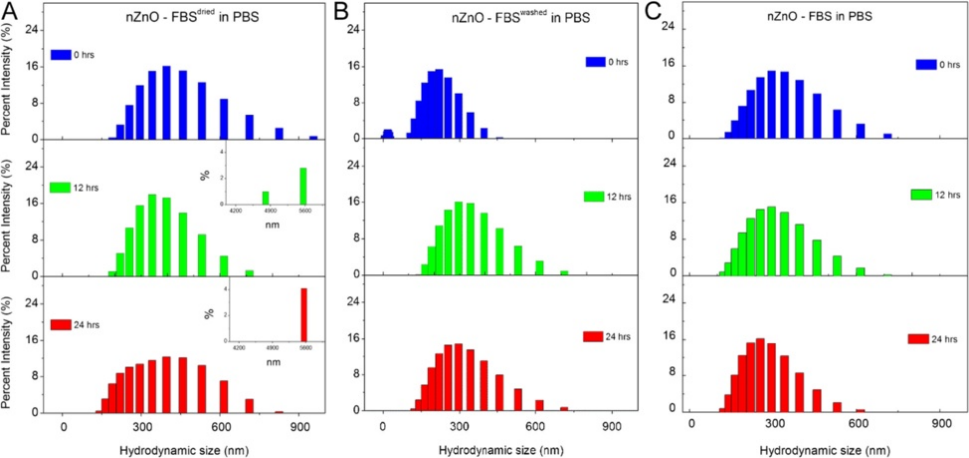
Hydrodynamic size distribution profiles at 0, 12, and 24 h. Histograms represent 25 mM stock dispersions prepared in PBS for **a** nZnO – FBS^dried^, **b** nZnO – FBS^washed^, and **c** nZnO – FBS

**Fig. 7:**
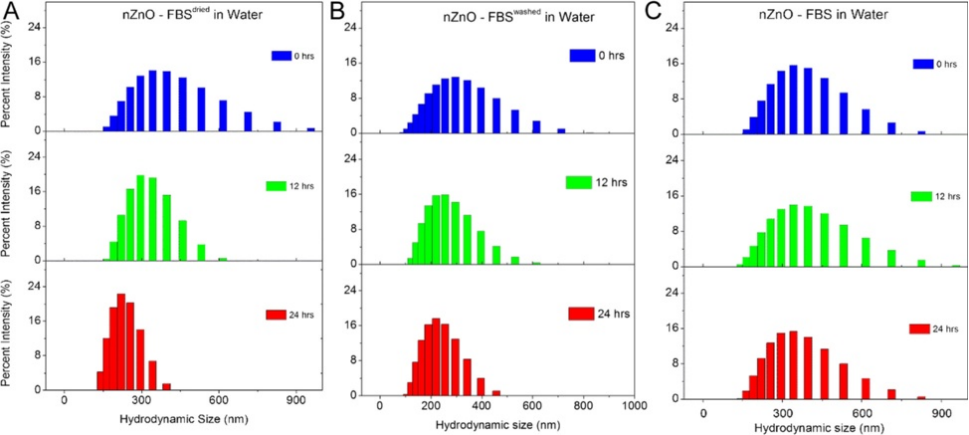
Hydrodynamic size distribution profiles at 0, 12, and 24 h. Histograms represent 25 mM stock dispersions prepared in water for **a** nZnO – FBS^dried^, **b** nZnO – FBS^washed^, and **c** nZnO – FBS

Further evidence of NP dispersion stability can be observed in the hydrodynamic size distribution profiles at various time intervals (Figs. 5, 6, and 7). Stable dispersions, such as those observed for the nanopure water (Fig. 5a), demonstrate similar size distribution trends across the evaluated time period. Strong agglomeration and sedimentation tendencies result in broader distributions initially as a result of immediate agglomerate formation, followed by narrower distributions as large agglomerates settle out of the dispersion. This trend is readily apparent for the PBS dispersion (Fig. 5b) and present to a lesser degree for the cellular media stock (Fig. 5c) which reflects the improved stability of the nZnO in RPMI-based cellular media, containing 10 % FBS, compared to the PBS stock. The size distribution trends observed for the nZnO – FBS^dried^, nZnO – FBS^washed^, and nZnO – FBS dispersions in PBS (Fig. 6) and water (Fig. 7) affirm the findings previously observed. Both the nZnO – FBS^dried^/PBS (Fig. 6a) and nZnO – FBS^dried^/water (Fig. 7a) dispersions begin to show dispersion instability at ~12 h which is readily apparent by 24 h. Taken together, the agglomeration profiles obtained from DLS and the sedimentation trends observed by UV-Vis spectrophotometry provide an accurate qualitative assessment of the NP dispersion stability.

**Fig. 8:**
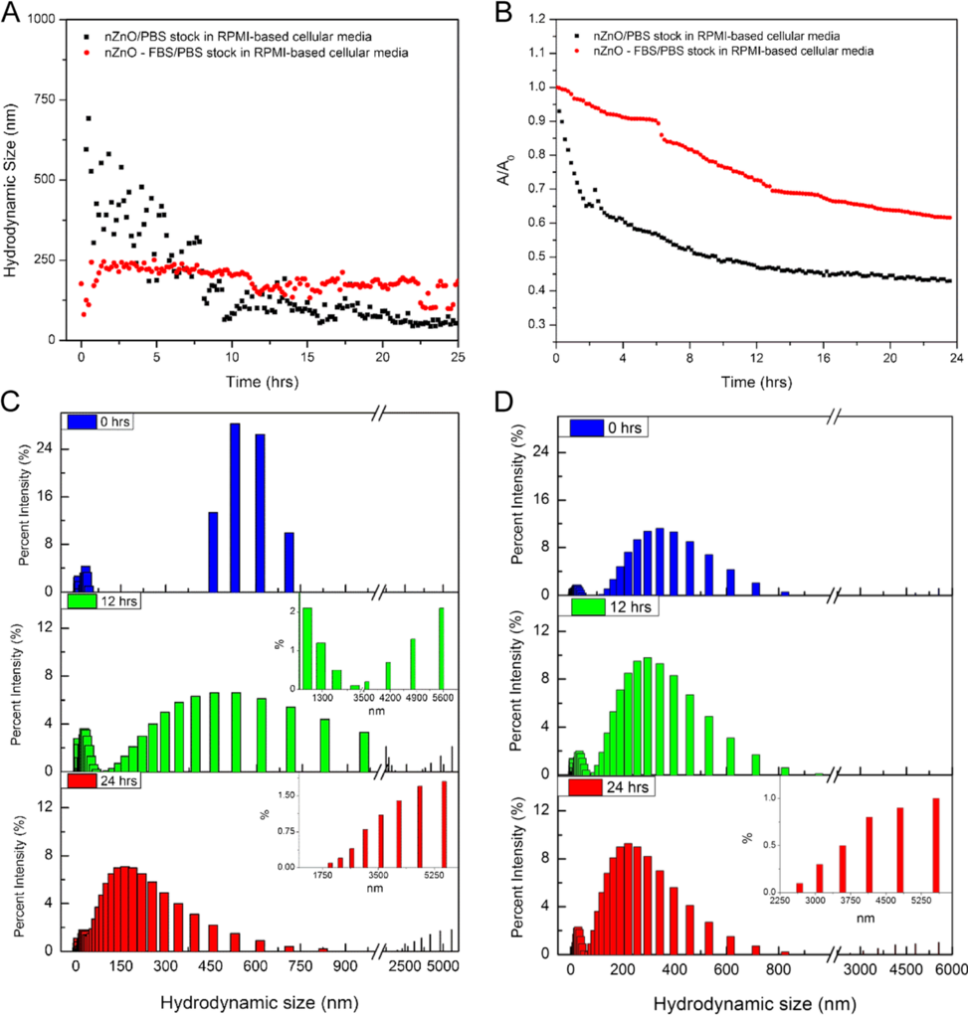
Dispersion stability analysis for nZnO/PBS and nZnO – FBS/PBS stock solutions introduced to RPMI-based cellular media at a concentration of 0.6 mM. **a** Average hydrodynamic size profiles for a 24-h time period. **b** Sedimentation profiles for a 24-h time period. Hydrodynamic size distribution profiles at 0, 12, and 24 h for **c** nZnO/PBS stock dispersion in RPMI-based cellular media and **d** nZnO – FBS^/^PBS stock dispersion in RPMI-based cellular media

After evaluating several stock dispersions independently, the behavior of the NPs was assessed under conditions used for cellular toxicity assays. In short, aliquots of the 25 mM nZnO/PBS and nZnO – FBS/PBS stock dispersions were introduced to RPMI-based cellular media at concentrations of 0.6 mM and the agglomeration and sedimentation tendencies of the NPs were observed for 24 h (Fig. 8). Analysis of the data collected under in vitro experimental conditions reveal that NPs dispersed in nZnO/PBS display strong agglomeration potential in the cellular media compared to those prepared using the nZnO – FBS/PBS stock. NPs in the dispersion prepared using the PBS stock formed large agglomerates immediately (>500 nm) (Fig. 8a, c), which precipitated rapidly from the cellular media (Fig. 8b), reducing the percent intensity and changing the maximum in the agglomerate size distribution (Fig. 8c). At the 12-h time point, the hydrodynamic size profile revealed a large distribution of NP sizes present within the solution with a 54 % decrease in the nZnO absorbance. Significant decreases in the hydrodynamic size distribution can be seen by the 24-h time point suggesting that large agglomerates had precipitated from the cellular media (Fig. 8a, c). The sedimentation profile (Fig. 8b) confirms that by 24 h nearly 60 % of the nZnO was no longer present in the cellular media. Comparatively, the experimental dispersion (0.6 mM) prepared from the nZnO – FBS/PBS stock exhibited considerably more size stability over time (Fig. 8a, d) resulting in less sedimentation behavior (39 %) by 24 h (Fig. 8b). In the context of in vitro cytotoxicity studies, these results imply that using FBS as a NP stabilizing agent prior to solution dispersion results in more NPs being bioavailable to interact with cells cultured in suspension. Although most of the ZnO NPs are in the 100–250 nm range at the 24-h time point, there are some significantly large agglomerates (3000–6000 nm) in the hydrodynamic size profiles (inset in Fig. 8d) of nZnO – FBS samples. However, their percent abundance is <5 % of the total number of observed aggregates at 24 h.

### Dissolution Studies

**Fig. 9:**
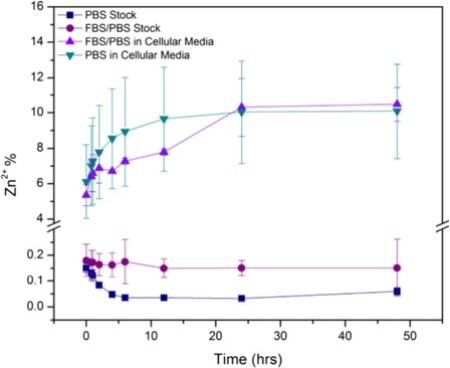
Extracellular dissolution of 6 mM nZnO/PBS and nZnO – FBS/PBS stock dispersions and 0.6 mM of those respective nZnO stock dispersions in RPMI-based cellular media. Zn^2+^ concentration is expressed as % Zn^2+^ (total dissolved Zn^2+^ measured by ICP-MS/total Zn^2+^ in sample) to normalize for concentration

Experiments were conducted to investigate if Zn^2+^ ions are released from the ZnO NP, when dispersed in stock solutions prepared at concentrations typically employed for downstream biological assays (6 mM of nZnO). Zn^2+^ dissolution was measured using a PBS stock dispersion and compared to the same after adding 10 % FBS to improve dispersion stability. Figure 9 shows the Zn^2+^ dissolution data (displayed as percentage of the total nZnO concentration in each solution as a function of time). Among these, the nZnO – FBS/PBS stock dispersion showed the highest Zn^2+^ dissolution (0.151 ± 0.029 % at 24 h) while the nZnO/PBS stock (0.033 ± 0.004 % at 24 h) showed significantly less Zn^2+^ ion release. These dissolution studies were then extended to samples prepared under actual experimental conditions (0.6 mM nZnO) used for the in vitro toxicity assay studies as previously described. This lowering of the nZnO concentration by 10-fold caused significant increases in percentage Zn^2+^ dissolution for both dispersion combinations, as shown in Fig. 9. Decreasing nZnO concentration in solution will decrease NP agglomeration tendencies and subsequently increase the interaction of the nanoparticles with the medium, resulting in more Zn^2+^ ion formation in the dispersion [63]. Interestingly, the FBS/PBS sample showed lower Zn^2+^ dissolution (8.668 ± 0.247 % at 24 h) than the pure PBS sample (10.041 ± 2.905 % at 24 h). Since FBS addition increases dispersion stability and reduces hydrodynamic size, an increase in the Zn^2+^ dissolution is expected if interaction of the NPs with the dispersed medium alone is the most important factor. The observed lower Zn^2+^ dissolution of the nZnO – FBS/PBS sample may be due to the presence of serum proteins from FBS on the nZnO surface, reducing direct NP-media interaction and thus protecting them from Zn^2+^ dissolution. Both FTIR and zeta potential measurements have shown that 10 % FBS addition produces a surface layer of serum proteins on the nZnO surface. Thus, the lower Zn^2+^ dissolution of nZnO – FBS/PBS dispersion (0.6 mM) is likely attributed to the serum coating of the nZnO and the resulting reduction in the NP-medium interactions. Another potential contributing factor in these systems is the propensity of free zinc ions to form insoluble zinc carbonate (ZnCO_3_) or zinc phosphate (Zn_3_(PO_4_)_2_) precipitates in biologically relevant solutions [61, 64]. Precipitation of these species may explain dissolution potential differences between the prepared nZnO/PBS and nZnO – FBS/PBS stock dispersions given the assumption that the FBS present in suspension may provide a protective function against nZnO and Zn^2+^/media interactions, thus reducing the amount of insoluble complexes formed and increasing the concentration of ionic Zn^2+^ [65]. However, if this were the sole contributing factor, the same dissolution pattern would be expected when the stock dispersions were introduced into the cellular media. The reversal of the dissolution potential trend going into the cellular media underscores the complex nature of nZnO dissolution in biologically relevant solutions. While our results appear to contradict a recent study by Hsiao and Huang [41] in which a much larger Zn^2+^ dissolution was observed when FBS-coated ZnO NPs were dispersed in DMEM media, these apparent discrepancies may be attributable to solution-related dissolution differences. Additional studies conducted in our laboratory have shown that nZnO dispersed in the RPMI-based cellular media employed in this study exhibit significantly less dissolution potential than nZnO dispersed in the DMEM used by Hsiao and Huang (data not shown). The decrease in ionic zinc in the RPMI-based media compared to DMEM could be attributed to increased precipitation of insoluble Zn_3_(PO_4_)_2_ in the phosphate-rich RPMI system [61, 65]. Furthermore, NP dissolution in the presence of serum proteins appears to be a competition between two processes. Serum proteins reduce agglomerate size allowing more interaction between NP surface and the dispersing medium. However, serum proteins will also attach to the nanocrystal surface effectively reducing the interaction of surface bound Zn ions and the medium.

### Reactive Oxygen Species

**Fig. 10:**
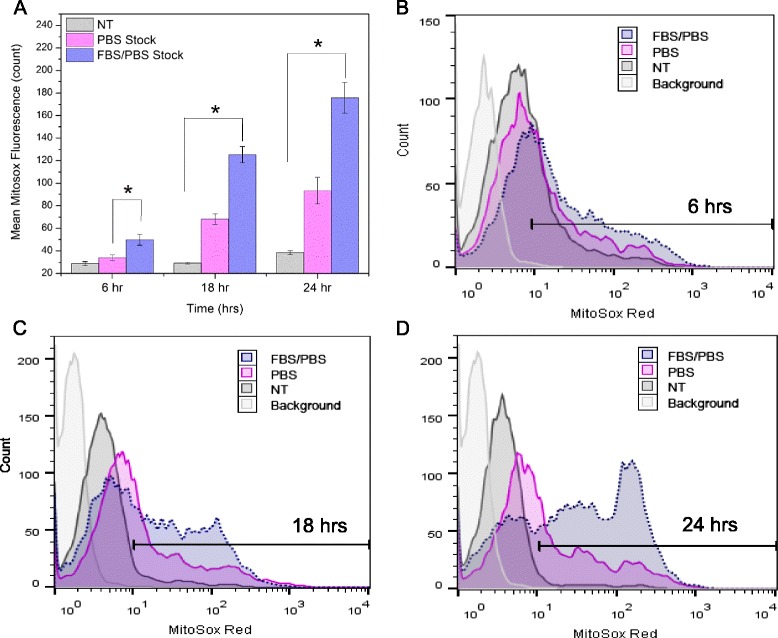
Cellular production of ROS following ZnO NP exposure. ROS generation was evaluated in Jurkat cells at 6, 18, and 24 h following treatment with nZnO/PBS (PBS) and nZnO – FBS/PBS (FBS/PBS) stock dispersions using MitoSOX red and flow cytometry (means ± standard error, minimum of *n* = 3). Cells were treated with 0.4 mM nZnO and stained with MitoSOX red and CD3 antibody after the desired time. **a** Mean MitoSOX fluorescence intensity for a non-treated control (NT) and nZnO and nZnO – FBS exposure. To assess relative increases in ROS following NP treatment, a marker (M1) was set so that background fluorescent in control samples (MitoSOX loaded/no NP) was between 1 and 3.5 %. Histograms represent **b** 6 h, **c** 18 h, and **d** 24 h

The potential role of intracellular reactive oxygen species (ROS) generation in the cytotoxicity of nZnO was demonstrated in our earlier reports [10, 15, 16] and through several other studies for a variety of cells types [66–68] by using fluorescent dyes to directly detect ROS. Since redox reactions at the NP surface are a primary driver in extracellular and intracellular ROS production, changes in the NP surface structure as a result of protein coating could change ROS generation and, in turn, also influence the cytotoxicity. To evaluate the amount of ROS produced by nZnO and nZnO – FBS, Jurkat cells were exposed to 0.4 mM ZnO for 6, 18, and 24 h and subsequently stained with MitoSOX Red. As Fig. 10 illustrates, appreciable differences exist between the amounts of ROS produced at 6 h (Fig. 10a, b), as measured by the mean MitoSOX fluorescence intensity (MFI), between the nZnO dispersion (PBS stock; 33.9 ± 2.66 MFI) versus the nZnO – FBS dispersion (FBS/PBS stock; 49.9 ± 4.98 MFI) representing a 47.2 % increase in the amount of NP-induced ROS present in cells treated with the nZnO – FBS dispersion. As the exposure time increases, the differences in the ROS production increase significantly. The greatest disparity, noted at 24 h (Fig. 10a, d), represents an 88.2 % increase in the amount of ROS induced by the nZnO – FBS over the nZnO dispersion. Given that the agglomerates present in the nZnO – FBS dispersion are significantly smaller and more bioavailable than those in the nZnO dispersion, more surface area to volume interactions are possible in the more stable nZnO-FBS which could result in the significant differences observed in the ROS induction. While many surface modifications, such as SiO_2_ coating, have demonstrated protective benefits such as less cytotoxicity and cellular stress-inducing ROS production [42, 66], FBS coating does not appear to impart protective benefits against ROS generation.

### Cellular Toxicity Studies

**Fig. 11:**
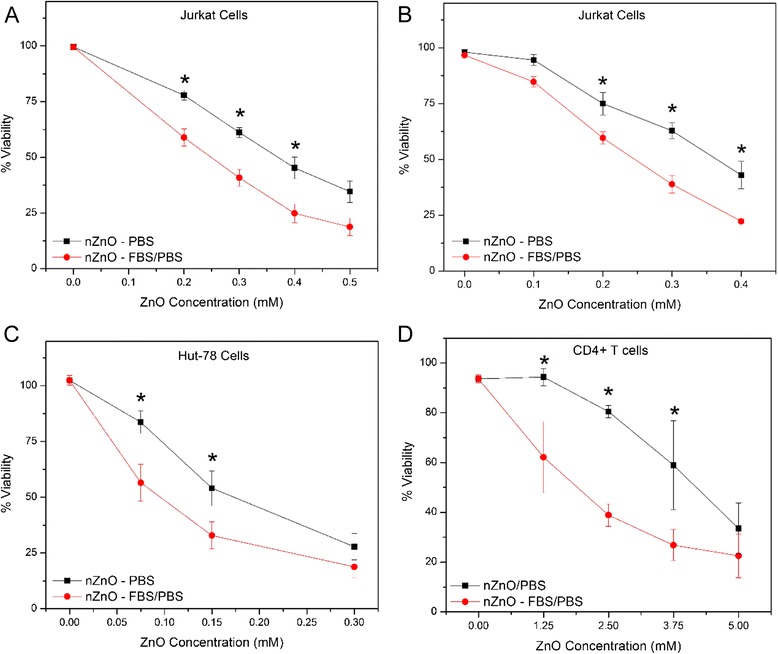
ZnO NP toxicity using nZnO/PBS and nZnO – FBS/PBS stock dispersions for 24 h on various suspension cell types. Cultures were treated concurrently with varying concentrations of ZnO NP for 24 h and cell viability was evaluated (means ± standard error, minimum of *n* = 3). Statistical analysis was performed using repeated measures analysis of variance and model-based means post hoc test (*p* < 0.05) with an asterisk denoting statistical significance. Jurkat cell viability was assessed using both **a** alamar blue staining or **b** flow cytometry and PI uptake to evaluate between assay variability and repeatability. **c** Hut-78 cell viability using alamar blue staining and **d** CD4+ T cell viability using flow cytometry and PI uptake

It has been previously shown in our laboratory that these nZnO synthesized in DEG media demonstrate strong cytotoxic effects against Jurkat leukemic and Hut-78 lymphoma T cell cancer lines [16]. Experiments were performed to determine if FBS coating and the resulting improvements in NP stability and bioavailability influence the cytotoxicity of NPs in these suspension cell models. Cells were exposed to increasing concentrations of freshly prepared nZnO/PBS or nZnO – FBS/PBS stock dispersions for 24 h. NP-induced toxicity was then determined using an alamar blue assay or through flow cytometry utilizing a T cell-specific fluorescent antibody and propidium iodide (PI) dye uptake [16]. As shown in Fig. 11, both types of cancer cells showed a significant increase in the NP-induced cytotoxicity (*p =* 0.0022 and *p* = 0.0488 for Jurkat and Hut-78 cells, respectively) when nZnO – FBS/PBS stock-treated cultures were employed, thus confirming the favorable role of 10 % FBS addition in the stock dispersion preparation. The IC_50_ values for Jurkat cells decreased from 0.39 ± 0.03 mM for nZnO/PBS dispersions to 0.27 ± 0.02 mM for the nZnO – FBS/PBS dispersion using alamar blue staining (Fig. 11a) and from 0.38 ± 0.03 mM for uncoated nZnO to 0.25 ± 0.004 mM for nZnO – FBS using flow cytometry and PI uptake (Fig. 11b). Similarly, the IC_50_ value for the Hut cells (Fig. 11c) also showed a decrease from 0.21 ± 0.03 mM when treated with the nZnO/PBS dispersion to 0.14 ± 0.02 mM when treated with the nZnO – FBS/PBS dispersion. These results clearly demonstrate that coating nZnO with FBS proteins and the resulting improved dispersion stability increases their toxic response to cancer cells by ~33 % for both cell types. Additionally, FBS coatings may also provide a potential approach to reduce the IC_50_ values of nZnO against cancer cells from their current millimolar range to more suitable ranges appropriate for therapeutic applications.

Our previous work has shown that ZnO NPs display some selectivity in their toxic response against cancerous T cells as the IC_50_ values observed for primary quiescent T cells under similar conditions are considerably higher. Similar toxicity studies using nZnO – FBS/PBS dispersions also showed stronger toxic responses (*p =* 0.0303) to resting CD4^+^ T lymphocytes (Fig. 11d). Here, the IC_50_ values decreased from 4.69 ± 0.85 mM when treated with nZnO/PBS dispersion to 2.43 ± 0.0.24 mM when nZnO – FBS/PBS dispersions were used. Thus, the effect of coating NP with serum proteins is evident in primary cells as well. While these results are consistent with our previously reported preferential cytotoxicity of nZnO to cancer cells, the therapeutic index of Hut cancer cells decreased from 22.6 for nZnO/PBS dispersions to 17.0 for the nZnO – FBS/PBS dispersion. Even with the modest decrease noted in the therapeutic index for the nZnO – FBS/PBS dispersion, the resulting therapeutic efficiency of the nZnO appears better than some commonly recognized traditional chemotherapeutic agents using comparable in vitro assays [16]. Thus, the protein interaction with nZnO increases their cytotoxicity in the suspension cell models evaluated, yet maintains some of the cancer cell selectivity previously observed.

**Fig. 12:**
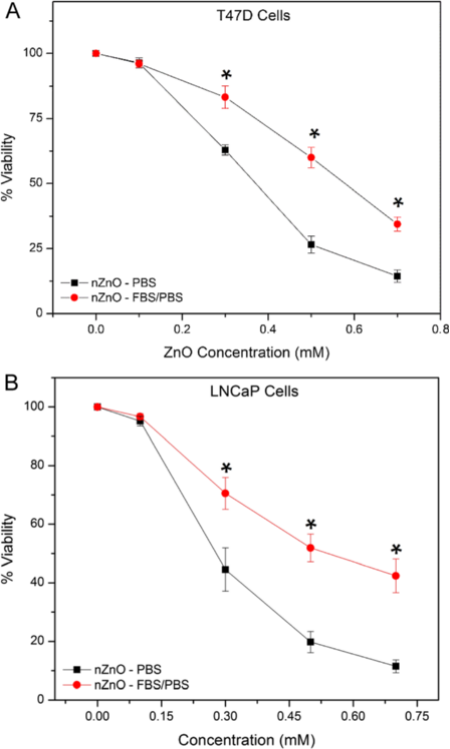
ZnO NP toxicity using varying concentrations of nZnO/PBS and nZnO – FBS/PBS stock dispersions for 24 h on adherent cell types. **a** T-47D epithelial mammary gland carcinoma and **b** LNCaP epithelial prostate cancer cells. Cultures were treated concurrently and cell viability was evaluated (means ± standard error, minimum of *n* = 3). Statistical analysis was performed using repeated measures analysis of variance and model-based means post hoc test (*p* < 0.05) with an asterisk denoting statistical significance

Some published studies have reported that coating nZnO with organic molecules or media protein components results in decreased cytotoxicity and ROS accumulation compared to uncoated nZnO [25, 38, 40–42, 69]. While these studies appear to contradict our findings, it should be noted that most of these reports evaluated adherent cells while the primary T cells and cancerous counterparts employed here are all grow as suspension cultures. In adherent cell cultures, poor dispersion stability results in faster sedimentation of NP agglomerates onto the cell monolayer. This rapid sedimentation may artificially lead to higher increases in NP-cell interactions [34] and the observed increased toxicity [39, 41, 42]. When the dispersion stability improves and sedimentation decreases with the addition of suitable molecules such as FBS, adherent cells will be exposed to lesser amounts of deposited NPs resulting in more accurate dosimetry kinetics, measured effective administered doses, and observed toxicity values, as reported by many recent studies [25, 38, 40–42, 69]. This possibility was confirmed by comparing the effects of nZnO/PBS and nZnO – FBS/PBS dispersions in two different adherent epithelial cancer cell lines, T-47D mammary gland carcinoma and LNCaP prostate cancer cell lines (Fig. 12). For both adherent cell lines, the nZnO – FBS/PBS-treated cells exhibited less toxicity when compared to the nZnO/PBS-treated cells (*p* = 0.0020 and *p* = 0.0008 for the T-47D and LNCaP cells, respectively). For the T-47D (Fig. 12a) cells, a 48.7 % increase in the IC_50_ was observed for the nZnO – FBS/PBS dispersions over the nZnO/PBS-treated cells (from 0.39 ± 0.02 mM for nZnO/PBS to 0.58 ± 0.01 mM for nZnO – FBS). Comparatively, the IC_50_ for the LNCaP cells (Fig. 12b) increased from 0.35 ± 0.02 mM nZnO/PBS-treated cells to 0.60 ± 0.06 mM for nZnO – FBS/PBS-treated cells representing a 71.4 % decrease in the toxic response.

In comparison to the suspension cell models, FBS coating leads to opposite effects in adherent cells. Suspension cells interact with fewer NPs in experimental conditions consisting of even minimally unstable dispersions since a portion of the original NP concentration sediments and may not be bioavailable for cellular interactions. This will skew toxicity assay results to suggest lower toxicity for suspension cells than expected. When the stability of the NP dispersion is improved by suitable molecular additives such as 10 % FBS (Fig. 11), the cytotoxicity increases significantly for both Jurkat and Hut cancer cells as well as for the primary T cell lymphocytes. More ZnO NPs are freely available in the stable NP dispersions to interact with more cells, thus resulting in the higher cytotoxicity observed in suspension cell models. The stability-induced differences between suspension and adherent cells underscore the importance of this work. The results from suspension cell studies alone would seem surprising considering other reports employing similar serum coatings describe decreases in NP-induced cytotoxicity in adherent cell lines suggesting that NP coating could be a potential means to control unwanted cytotoxicity [39]. One such study, Hsiao and Huang [41], demonstrated that 5–10 % FBS coating on ZnO NPs increased dispersion stability and Zn^2+^ ion release, and decreased the NP-induced cytotoxicity. They hypothesized that this reduction in cytotoxicity in the serum-coated NPs was a result of serum-induced increases in cell growth and resulting decreases in nZnO dosage-per-cell ratios. Additionally, these authors [41] showed larger Zn^2+^ release in their experiments with FBS-coated ZnO thus negating Zn^2+^ release as a primary contributing mechanism to nZnO cytotoxicity. In this present study, FBS coating appears to decrease the Zn^2+^ release while significantly increasing NP-induced cytotoxicity for the suspension cell model when compared to uncoated nZnO. With the addition of the 10 % FBS to our phosphate-rich stock dispersion, it is plausible that decreases in formation of toxic insoluble Zn_3_(PO_4_)_2_ precipitates [70] could translate to perceptible decreases in the NP-induced cytotoxicity observed for nZnO – FBS/PBS treatment. However, decreases in Zn_3_(PO_4_)_2_ formation would also result in measured increases in Zn^2+^ release which was not observed. Therefore, extracellular Zn^2+^ dissolution is unlikely to be a primary factor for the increased cytotoxicity noted for our nZnO – FBS NPs. It is clear from FTIR and zeta potential studies that FBS molecules bind to the ZnO NP surface. This modification of the NP surface influences their interaction with other NPs (and on agglomerate formation and sedimentation effects) as well as with cells.

### Modeling and Dosimetry Studies

Given the toxicity trend differences noted for the differing cell models, mathematical models were investigated as a potential method to confirm the observed trends and to model the dosimetry kinetics for both the investigated stock dispersions (nZnO/PBS versus nZnO – FBS/PBS) and the different cellular models. All of the investigated experimental parameters of dispersion stability can be applied to the in vitro sedimentation, diffusion, and dosimetry (ISDD) computational models, put forth by Hinderliter et al. (2010), to predict the transport properties of the NPs in various experimental conditions [43]. The NP and experimental parameters which most influence the NP transport properties include hydrodynamic size (*d*_H_), agglomeration state, effective particle density (*ρ*_e_), and the media height in the cell well (*L*) [43]. Central to the effective dosimetry determination in the ISDD model is the calculation of a NP deposition factor *α* (h^−1^), Eq. 2 in “Experimental Details”, which interrelates the diffusion rate (*D*), sedimentation velocity (*V*), and media height [32, 33, 43, 44]. While the diffusion rate is primarily dependent upon the hydrodynamic size, the sedimentation velocity depends upon both the hydrodynamic size and the effective particle density occupied by NP agglomerates [43, 44]. The effective particle density (*ρ*_e_) can be approximated in the ISDD model (Eq. 7) using the hydrodynamic size-dependent agglomerate porosity parameter (*ε*_agg_) [43] or can be determined experimentally (Eq. 1) as a part of the Harvard Volume Centrifugation Method (VCM) [33, 43]. The Harvard VCM employs an experimental determination of NP agglomerate volumes to directly determine effective particle density. Furthermore, the VCM has been modified to incorporate mass lost due to the dissolution of soluble or partially soluble NPs such as ZnO [32, 33].

**Fig. 13:**
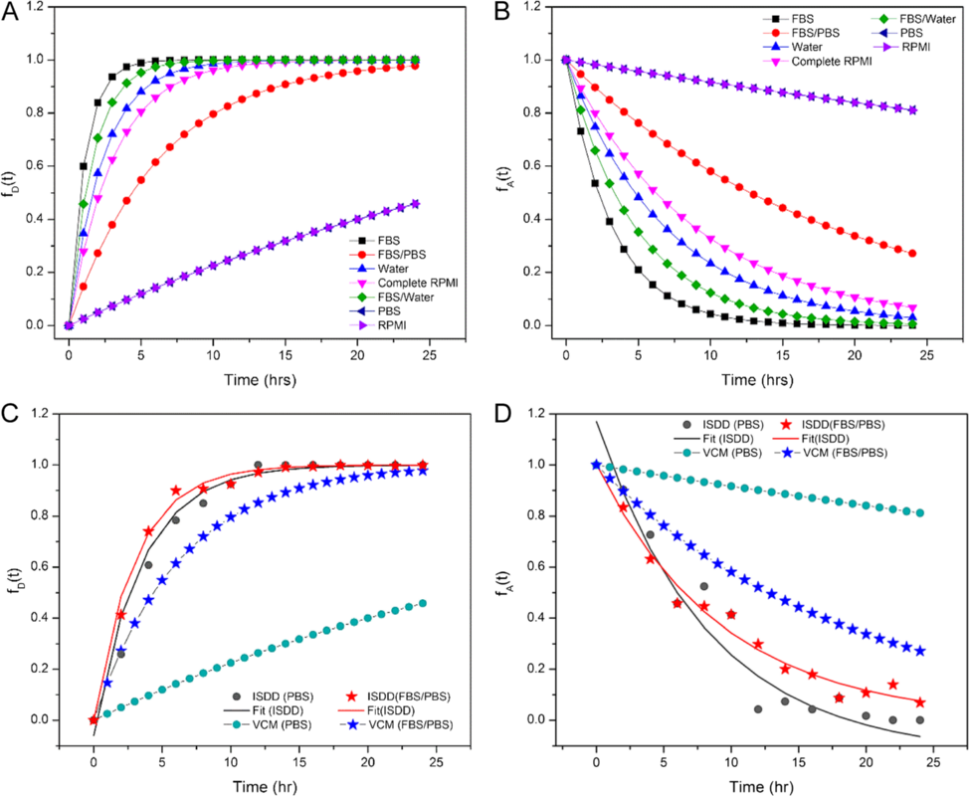
Dosimetry curves for various stock dispersions introduced to RPMI-based cellular media at a concentration of 1.25 mM. **a** Curves constructed using *α* values calculated for 24-well plates and representing the fraction of NP deposited [*f*_*D*_(*t*)] to an adherent cell as a function of time. **b** Curves constructed using *α* values calculated for 96-well plates and representing the fraction of NPs available [*f*_*A*_(*t*)] to cells grown in suspension as a function of time. Dosimetry curves constructed using both the Harvard VCM and ISDD models for both the nZnO/PBS and nZnO – FBS/PBS stocks representing **c** NP deposition [*f*_*D*_(*t*)] and **d** NP availability [*f*_*A*_(*t*)]

To determine dosimetry curves for various experimental conditions, 25 mM dispersions were prepared and dispersed in complete cellular media at a concentration of 1.25 mM and the effective density (*ρ*_e_) determined using Harvard VCM (Table 1) [33]. From the calculated deposition factor, *α* (h^−1^), dosimetry curves were constructed (Fig. 13) as described in Cohen et al. (2014) [33]. Originally introduced as a model for adherent cell culture, the deposition fraction curve [*f*_*D*_(*t*)] (Eq. 3; Fig. 13a) depicts the fraction of NPs that are deposited upon an adherent cell layer as a function of time [32, 33]. However, the same *α* values may be used to model the fraction of NPs that remain bioavailable [*f*_*A*_(*t*)] as a function of time for suspension cells (Eq. 5; Fig. 13b). Based on these curves, a *t*_90_ value can be calculated to make accurate determinations of effective NP dose either deposited on an adherent cell layer or bioavailable to suspension cells at desired time points.

**Table 1:** Delivered dose metrics

Stock dispersion in complete RPMI	Zeta potential (mV)	*d*_H_ (nm)	*ρ*_e_ (g/cm^3^)	96-well plate		24-well plate	
				(*L* = 6.6 mm)		(*L* = 2.25 mm)	
				*α* (h^−1^)	*t*_90_ (h)	*α* (h^−1^)	*t*_90_ (h)
Water	−16.1 ± 1.45	182.5	2.021	0.1453	15.6	0.4261	5.4
FBS/Water	−15.1 ± 0.81	185.1	1.692	0.2085	11.1	0.6116	3.8
FBS	−13.9 ± 0.40	185.5	1.464	0.3121	7.4	0.9154	2.5
PBS	−8.1 ± 1.93	663	1.879	0.0087	264.9	0.0255	90.3
FBS/PBS	−9.8 ± 2.08	322	1.743	0.0543	42.4	0.1593	14.5
RPMI	−14.7 ± 1.46	770.5	1.623	0.0087	263.8	0.0256	89.9
Complete RPMI	−13.6 ± 2.63	244.4	1.682	0.1116	20.6	0.3273	7.1

Delivered dose metrics: *ρ*_e_ is the agglomerate effective density (g/cm^3^); *d*_H_ is the hydrodynamic diameter (nm); *L* is the media height; *α* is the deposition fraction (h^−1^); *t*_90_ is the time required for delivery of 90 % of administered dose (h); and *α* (h^−1^) and *t*_90_ (h) values are designated for each well plate employed (96-well plate for suspension cells and 24-well plate for adherent cells)

*FBS* fetal bovine serum, *PBS* phosphate buffered saline, *RPMI* Roswell Park Memorial Institute medium

The influence of FBS on the dosimetry kinetics is evident in an analysis of the experimentally stable water, FBS/water, and FBS stock dispersions introduced to the RPMI-based cellular media. Given the dependence of agglomerate behavior and effective density (*ρ*_e_) on the hydrodynamic size of NPs in dispersion, the water (182.5 nm), FBS/water (185.1 nm), and FBS (185.5 nm) stock dispersions should exhibit similar dosimetry kinetics. However, the effective density (*ρ*_e_) of the NPs in the three dispersions decreased with increasing amounts of FBS in the stock preparation thus increasing the calculated deposition factor. These disparities are a direct result of measured increases in the effective volume of the NP pellet with increasing amounts of FBS in solution. So while the similarities in the hydrodynamic diameters of these three dispersions imply consistent NP diffusion rates (Eq. 9), the differing effective volume measurements suggest different NP sedimentation velocities (Eq. 8) [32]. Therefore, even though all three dispersions demonstrate dispersion stability experimentally, the effective volume occupied by the resulting protein corona in the NP agglomerates decreases the sedimentation velocities of the NPs predicted by the model, thus increasing the deposition factor and shifting the dosimetry kinetics.

Interestingly, the dose metrics obtained for the unstable stock dispersions of PBS and RPMI suggest dosimetry trends opposite to what would be expected considering the experimentally determined agglomeration and sedimentation profiles. However, the VCM dosimetry model assumes that the dispersions used to obtain the experimental *α* values are stable across the time frames defined by the model. As this is not the case with the PBS and RPMI stock dispersions, these obtained dosimetry curves cannot be used to accurately model the dosimetry kinetics under these conditions. To model potentially unstable dispersions, it is necessary to calculate *α* values using experimentally determined stability parameters such as hydrodynamic size at multiple time points. For this, we employed the ISDD model. This model approximates effective particle density utilizing the hydrodynamic size-dependent parameter *ε*_agg_. While the approximation of the *ρ*_e_ employed in this model lacks the experimental accuracy achieved for *ρ*_e_ measurements afforded by VCM, the ISDD model does allow for time-resolved approximation of NP transport parameters within unstable dispersions. Employing the ISDD model, *α* values were obtained using time-resolved *d*_H_ values and the dosimetry curves for both the nZnO/PBS and nZnO – FBS/PBS stock dispersions in RPMI-based cellular media were modeled for several time points from 0 to 24 h. These time-resolved points were compiled to generate ISDD curves which were then fit to both the *f*_*D*_(*t*) and *f*_*A*_(*t*) mathematical models to obtain *α* values for each representative graph (Fig. 13c, d).

**Fig. 14:**
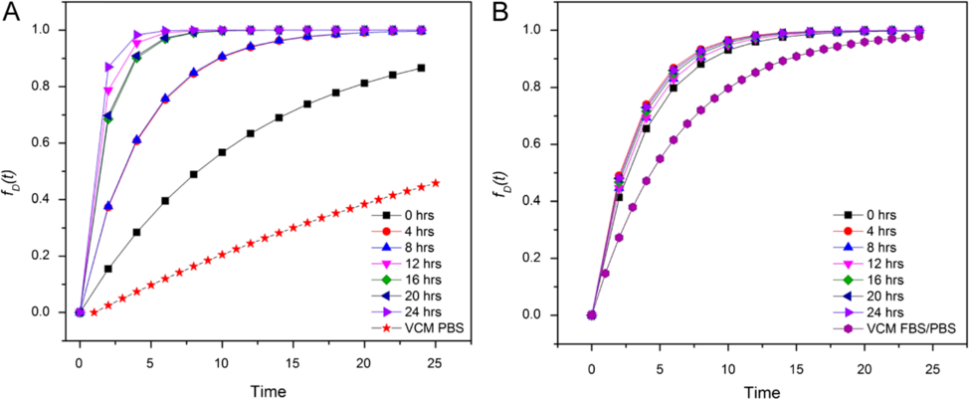
Dosimetry curves constructed using both the Harvard VCM and ISDD models for nZnO/PBS and nZnO – FBS/PBS stock dispersions introduced to RPMI-based cellular media at a concentration of 1.25 mM. Curves were constructed using *α* values calculated for 24-well plates at various time points (ISDD) and Harvard VCM *α* values (Table 1). These curves represent the fraction of NPs deposited [*f*_*D*_(*t*)] to an adherent cell layer as a function of time for **a** nZnO – PBS stock dispersions and **b** nZnO – FBS/PBS stock dispersions

For the unstable nZnO/PBS stock, the ISDD *α* values of 0.290 for adherent cell model conditions and 0.120 for the suspension cell models were significantly higher (1037 and 1279 % increases for adherent and suspension cell models, respectively) than those obtained using the Harvard VCM (Table 1). The ISDD modeled *t*_90_ values of 7.94 (adherent cells) and 19.12 (suspension cells) were more consistent with the experimental stability data discussed earlier. Applying the same model to the stable nZnO – FBS/PBS stock dispersion, yielded *α* values (0.331 and 0.107 for adherent and suspension cell models, respectively) more closely aligned to the VCM determined values (Table 1) with percent increases of 107 and 97 % for adherent and suspension cell models, respectively. Surprisingly, the ISDD modeled curves for the nZnO/PBS (unstable) and nZnO – FBS/PBS (stable) dispersions imply similar dosimetry kinetics for both cellular models and, if considered without experimentally derived stability profiles (e.g., average hydrodynamic size profiles, size distribution histograms, and sedimentation analysis), would not reflect the true dosimetry for unstable stock dispersions. While the ISDD-derived *f*_*D*_(*t*) curve for the unstable nZnO/PBS stock dispersion (Fig. 13c) at face value implies stable dosimetry kinetics, the individual ISDD curves at each time point reflect the unstable nature of the nZnO/PBS dispersion (Fig. 14a). As a result, using either mathematical model to predict the effective dose for this dispersion would not be advisable. For the stable nZnO – FBS/PBS dispersion, both the VCM and ISDD models (Figs. 13 and 14b) depict stable dosimetry kinetics for both suspension and adherent cell models. For adherent cells, the *t*_90_ value of 14.5 h (Table 1; VCM) suggests 90 % of the NPs in suspension will come in contact with and potentially interact with the cell layer by the 24-h time point indicating that the experimentally observed toxicity values are an accurate assessment of the in vitro toxicity for adherent cell types. Mathematical models for stable dispersions in suspension cells may provide less definitive information regarding effective dose. While these models can accurately predict the amount of NPs remaining in dispersion as a function of time [*f*_*A*_(*t*)], the models fail to account for NP-cellular interactions that will occur within the suspension culture before sedimentation processes remove NPs from the dispersion. Better mathematical models are needed to predict the fate and transport of NPs in suspension cell models that include NP sedimentation rates as well as time-resolved NP-cellular interactions. Taken together, these observations underscore the importance of time-dependent stability studies for NP stock dispersions prior to the implementation of fate and transport dosimetry models and downstream experimental applications.

## Conclusions

Studies reported in this work show that ZnO nanoparticles form very large agglomerates with hydrodynamic sizes in the 300 to 20,000 nm range which and depend primarily on the solution composition used. Presence of such agglomerates leads to sedimentation of a significant portion of the nanoparticles which increases in time. For adherent cells, this will increase the effective NP concentration interacting with the cells at very early time points and may cause unusually large and potentially inaccurate in vitro toxicity measurements. This was demonstrated using two adherent epithelial cell lines (T-47D and LNCaP) with both cell types experiencing significant decreases in NP-mediated cell death with the more stable NP stock dispersions. Likewise, this sedimentation process will reduce the effective NP concentration remaining in the solution medium. Subsequent investigations using suspension cells showed that this phenomenon results in an opposite trend due to the reduction in the number of nanocrystals interacting with the suspension cells and, consequently, a lower than actual toxic response is obtained. Using a 10 % FBS/PBS stock to disperse the same nZnO, the IC_50_ values for Jurkat (Hut) cancer cells decreased from 0.39 mM (0.21 mM) for nZnO/PBS-treated cells to 0.27 mM (0.14 mM) for the nZnO – FBS/PBS dispersion. Their normal counterparts (CD4+ T lymphocytes) also showed a reduction in IC_50_ with 10 % FBS addition. Its IC_50_ decreased from 4.69 mM for nZnO/PBS dispersions to 2.43 mM when nZnO – FBS/PBS stock was used. On the other hand, treating the adherent cells T-47D and LNCaP using the nZnO – FBS/PBS dispersion resulted in 48.7 and 71.4 % reductions, respectively, in the NP-induced toxic response compared to nZnO/PBS dispersion. Surface modification of these nZnO using serum proteins seems to be a suitable approach to obtain stable dispersions and accurate cytotoxicity values, thus improving their utility in therapeutic applications.

This study has shown the important role of serum proteins in modifying the ZnO nanocrystal surface resulting in the formation of considerably smaller agglomerates and stable NP dispersions. Furthermore, these FBS surface coatings influenced Zn^2+^ release from the nanocrystals and induced changes in the mitochondrial function of the cells as evidenced by the increased ROS production in the nZnO – FBS/PBS-treated cells. Given these findings, it is readily apparent that the addition of serum proteins adds a level of complexity to our experimental understanding of NP-cell interactions. Further studies are needed to examine how FBS coatings change NP-cellular surface interactions, influence cellular uptake mechanisms, and modify molecular interactions with other extracellular matrix components. Additionally, these studies should be extended to include newer 3D cellular culture models which often employ complex biomolecular solution systems or molecular scaffolds to better mimic in vivo-like environments for mechanistic biological assays [71, 72]. These complex culture systems will influence the formation of the NP biomolecular corona and change the diffusion and sedimentation rates for NP dispersions especially those containing additional serum proteins. Furthermore, 3D culture systems alter cellular growth patterns, metabolism, genetic expression, and phenotype organization changing NP-induced cellular responses which could be further influenced by the addition of NP coating materials [72, 73]. Finally, these studies should be extended into other cell types. Molecular coatings are often considered as a means to achieve safer nZnO structures, yet little is known about how these molecular coatings will influence NP-cellular interactions with endothelial cells [18, 27, 74] and other primary immune system cells [10].
